# Social and Regional Factors Predict the Likelihood of Admission to a Nursing Home After Acute Hospital Stay in Older People With Chronic Health Conditions: A Multilevel Analysis Using Routinely Collected Hospital and Census Data in Switzerland

**DOI:** 10.3389/fpubh.2022.871778

**Published:** 2022-05-09

**Authors:** Nicole Bachmann, Andrea Zumbrunn, Lucy Bayer-Oglesby

**Affiliations:** Institute for Social Work and Health, School of Social Work, University of Applied Sciences and Arts Northwestern Switzerland, Olten, Switzerland

**Keywords:** older age, chronic health conditions, hospital discharge, nursing home, social inequality, regional inequality, multilevel analysis, Switzerland

## Abstract

If hospitalization becomes inevitable in the course of a chronic disease, discharge from acute hospital care in older persons is often associated with temporary or persistent frailty, functional limitations and the need for help with daily activities. Thus, acute hospitalization represents a particularly vulnerable phase of transient dependency on social support and health care. This study examines how social and regional inequality affect the decision for an institutionalization after acute hospital discharge in Switzerland. The current analysis uses routinely collected inpatient data from all Swiss acute hospitals that was linked on the individual level with Swiss census data. The study sample included 60,209 patients 75 years old and older living still at a private home and being hospitalized due to a chronic health condition in 199 hospitals between 2010 and 2016. Random intercept multilevel logistic regression was used to assess the impact of social and regional factors on the odds of a nursing home admission after hospital discharge. Results show that 7.8% of all patients were admitted directly to a nursing home after hospital discharge. We found significant effects of education level (compulsory vs. tertiary education OR = 1.16 (95% CI: 1.03–1.30), insurance class (compulsory vs. private insurance OR = 1.24 (95% CI: 1.09–1.41), living alone vs. living with others (OR = 1.64; 95% CI: 1.53–1.76) and language regions (French vs. German speaking part: OR = 0.54; 95% CI: 0.37–0.80) on the odds of nursing home admission in a model adjusted for age, gender, nationality, health status, year of hospitalization and hospital-level variance. The language regions moderated the effect of education and insurance class but not of living alone. This study shows that acute hospital discharge in older age is a critical moment of transient dependency especially for socially disadvantaged patients. Social and health care should work coordinated together to avoid unnecessary institutionalizations.

## Introduction

Worldwide countries face the challenge of providing adequate, affordable and patient-centered health and social care to their aging populations (1). Most people wish to age healthily, live an independent and socially integrated life in their communities and stay at home, if possible till the end of their life (2, 3). “Aging in place” integrated in one's community is also considered as key element of the quality of life in old age (4). However, the majority of the older persons are suffering from one or multiple chronic health conditions (CHC) e.g., diabetes, cardiovascular diseases or depression (5) and an increasing number of older people with functional limitations need health care and support with the activities of daily living (6, 7). Coping successfully with CHC in the sense of stabilizing the course of the disease, preventing exacerbation and avoidable hospitalizations requires a high degree of self-management, personal and social resources as well as access to adequate health care (8, 9).

If a hospitalization becomes inevitable in the course of a chronic disease, discharge from acute hospital care in older individuals is often associated with temporary or persistent frailty, functional limitations and the need for help with daily activities. Thus, this represents a particularly vulnerable phase of transient dependency on social support and health care. So, after an acute hospital stay a critical question for the older patient, his or her family and for the hospital discharge team is whether to discharge to the patients' home or to a nursing home (2, 10). Hospital discharge decision-making is described as a complex process involving stakeholders with a range of expertise, experience and perspectives. The older patient's voice is often not included enough in this process, especially under the time pressure of shortened length of stay (11, 12). Being transferred directly from acute hospital into long-term care is a common pathway, but is seen critical (13). Nursing home admission, often termed institutionalization, is a significant life event for older persons and often associated with negative outcomes such as restricted quality of life and loss of social network as well as a high burden for public and private finances (14). It may often be unavoidable, especially in the case of cognitive impairment, but should not take place unnecessarily or prematurely.

The risk of suffering from one or multiple CHC follows a social pattern. The lower the socioeconomic status (SES) of an individual, the higher the risk of chronic diseases and multimorbidity such as mental health disorders, diabetes, chronic respiratory diseases or cardiovascular diseases (15, 16). Several studies have found that the social context of a person's life determines not only the risk of exposure and the degree of susceptibility but also the course and outcome of a disease depending on the capability to cope with the disease (8, 17, 18). However self-management of CHC is too often discussed from a purely individualistic perspective, ignoring the social and cultural context in which this process happens (19, 20). “Central to these critiques is a hyper-individualistic conception of patients as autonomous self-regulating subjects making self-serving decisions” (19).

The Commission on Social Determinants on Health (CSDH) set up by the WHO published a conceptual framework to explain the complexity of the different impact levels and pathways between social aspects and health (21). In this framework they distinguish between (1) *structural determinants on the macro-level* (socioeconomic and political context) which generate and maintain social hierarchies and defining socioeconomic positions of groups and individuals and (2) *intermediary determinants on the meso-level* with categories such as material circumstances, behavioral and biological factors, psychosocial factors as well as the health care system. The structural determinants include the characteristic of the welfare state, which is postulated to have a significant impact on social inequality in health by framing the type of health care system in a society and in mediating the effects of the social determinants on health. Applying this concept to the goal of a high quality of life and “aging in place” it can be concluded that intermediary determinants have an impact on the risk of chronic diseases and functional limitations that make it difficult for socially disadvantaged groups to live independently in old age. Structural determinants cause these disadvantages, but, at the same time, may mitigate them with appropriate interventions (e.g., access to community services and care at home for all).

Beckfield et al. (22) propose an institutional theory of welfare states on the distribution of health in a population. They highlight the impact of “meso-level rule-like arrangements” (e.g., neighborhood resources, the local health care system) and of macro-level institutions (e.g., access to social and health care systems) “to create winners and losers in social life” and in this way determine social inequalities in health (p. 231).

Although the question of social predictors of non-home discharge or new institutionalizations after a hospital discharge is of great individual and societal importance, surprisingly little research exists to date on this topic. Several studies analyze the decision-making process from a hospital's perspective showing the relevance of hospital-level factors like quality of the discharge management. But these studies do not focus on the social situation of the patients [e.g., (12, 23, 24)]. There exists a broad range of studies as well as meta-analyses on the question of social predictors of nursing home admissions or institutionalizations in the general population (10, 14, 25–28). In a meta-analysis including 77 studies analyzing the predictors of nursing home admission in the general population of the USA, Gaugler et al. (14) found strong evidence, that the presence of a spouse reduces and being white increases the likelihood of living in a nursing home, independent of health status and demographic variables; for living alone they found no clear evidence. A second meta-analysis including 36 studies from different developed countries found strong evidence for a higher likelihood of living in a nursing home among persons with limited financial resources. They found moderate evidence for an association with a poor social network and inconclusive evidence for such an association with living alone and low education (10). However, there are few studies analyzing the social predictors of new institutionalization after hospital discharge. Gilbert et al. (29) studied the likelihood of admission to a nursing home after fall-related hospitalizations in England. They included age, gender, comorbidity level (Charlson Comorbity Index) and area deprivation (rurality, ethnicity, and deprivation index) in their model and found a higher risk associated with highest age, severe comorbidity and living in a non-deprived all-white area. The last result may be surprising, but it is in line with a broad consensus in the international literature, that migration background is negatively associated with the use of nursing homes (27). Agosti et al. (30) analyzed the association between the likelihood of home or non-home discharge in 1,849 patients in Italy and the question of living alone or with others. They controlled for age, gender, a range of health indicators including cognitive impairment and functional limitation and found living alone to be an important predictor of non-home discharge. In another study in Italy, Marengoni et al. (31) studied the allocation of the place of residence after the discharge of 830 patients admitted to an acute geriatric ward in association with living alone, having a caregiver, multimorbidity, physical functioning, and cognitive status. They only found functional status to be a significant predictor of discharge to nursing homes but given the small N of cases admitted to nursing homes (*N* = 23), this result may not be seen as conclusive. Harrison et al. (2) described the characteristics of patients entering a nursing home after hospitalization in a retrospective cohort study in one hospital in Scotland. They found that people discharged to nursing homes were predominantly female, widowed, older and living alone. However these were only descriptive results which were not adjusted for covariates such as health status, etc.

For many countries (e.g., the USA, Canada, UK, Germany) substantial regional variations in permanent placements in nursing homes are reported and seen as an indicator for local disparity in access and effectiveness of health and social care for older persons to promote and maintain their independence (32–36). In conclusion, our review identified few studies taking a comprehensive approach to analyzing the question of social inequality in nursing home admission after hospital discharge and few studies using representative samples. Two meta-analyses investigating the likelihood of institutionalization in the whole population found inconclusive evidence for social determinants. The few studies focusing on the phase after a hospital discharge are often restricted to only one aspect of the social situation or only one health condition (e.g., fall-related health problems). There is also the problem of limited explanatory power due to methodological limitations. To the best of our knowledge and in accordance with Harrison et al. (2) we conclude that the topic of social inequalities in admission to nursing homes after acute hospitalization in older people are poorly researched to date.

**Figure 1:**
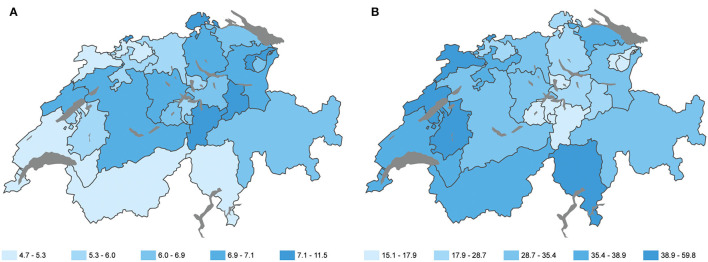
Nursing home places and use of ambulant long-term care in the language regions. **(A)** Number of nursing home places per 1,000 individuals 65 years old and more. **(B)** Number of people using ambulant home care (SPITEX) per 1,000 individuals in the cantons of Switzerland 2016 (Swiss Health Obervatory, 2021; reprinted with permission by Swiss Health Observatory).

In Switzerland, the federal legislation determines the basic framework of social and health insurance, but the 26 cantons are responsible for the conceptualization and implementation of old age policy and inpatient acute and long-term care. This leads to considerable regional disparities regarding access to specific health care for older people, including palliative care (37–39). A total of 154,634 individuals 65 years old and older were living in a nursing home in Switzerland in 2018, corresponding to a rate of 70.5 women and 33.4 men per 1,000 individuals in this age group. This proportion rises markedly with increasing age, with 1.5% of the population between 65 and 79 years old and 15% of the population above 79 years (40). There are 60.9 nursing home places per 1,000 individuals 65 years old and older in Switzerland, with substantial cantonal disparities, ranging from 46 places (Canton Wallis) to 101 places (Canton Appenzell Ausserrhoden) per 1,000 individuals in this age group (see also Figure 1A). The disparity in health care infrastructure for older persons is reflected in Figures 1A,B showing the density of nursing home places and the use of ambulant home care (SPITEX) in the cantons of Switzerland.

Based on cluster analyses of the data of 29 OECD countries to classify the different healthcare systems, Reibling et al. (41) conclude that the healthcare system in Switzerland is unique in Europe and forms a cluster with the USA only, portrayed as systems with high supply and low public but high private (out-of-pocket) expenditures. All residents in Switzerland have a compulsory basic health insurance, but this insurance class does not cover all services needed. For example dental care or household assistance after a hospital discharge are not covered. These services are covered by private and semi-private supplementary insurance only. In addition, there is a high proportion of cost-sharing. It is therefore not surprising that an outstanding high proportion of people in Switzerland experience financial barriers to health care: 22% of adults (31% of low-income adults, defined as member of households with an income less than half of the median household income) reported cost-related access problems to medical care in the preceding 12 months. This rate is at least twice as high as in countries such as Germany, the Netherlands, Sweden or the UK (42).

The aim of the present study is to analyze whether there are social inequalities in the likelihood of admission to nursing homes after an acute hospital stay in the older population with chronic conditions. In accordance with the CSDH framework, we will focus on intermediary social determinants such as education level, income and social support and at the same time on disparities between the language regions as indicators for the impact of structural determinants on the macro-level, while controlling for hospital-level characteristics. Based on the literature on the cumulative effect of multiple social disadvantages and exposures (43, 44), we want to analyze possible interaction effects between the three social predictors on the risk of nursing home admission. Lastly, we also test for a possible moderator effect of the regional characteristics on the relationship between social predictors and outcome, by including interaction terms between language regions and social predictor variables.

We hypothesize that: (a) people high in resources (education, supplementary insurance and social support) are less likely to be institutionalized after acute hospital stay independent of gender, age, nationality, type of chronic condition and comorbidities as well as clustering effects on hospital-level; (b) the likelihood depends on the language region; (c) the language region moderates the relationship between social determinants and the likelihood of new institutionalizations.

## Method

### Data Sources and Linkage Procedure

The database used for this study was established within the project “Social Inequalities and Hospitalisations (SIHOS).” SIHOS is part of the National Research Program 74 “Smarter Health Care,” which is promoting innovative health services research and helping to tackle the practical challenges of caring for the chronically ill in Switzerland.^1^ Thanks to the partial revision of the Statistical Surveys Ordinance in the year 2014, different public census data and administrative hospital data could be linked for the first time on an individual level in Switzerland. The SIHOS database was created to include demographic and socioeconomic variables as well as information on hospital stays and stays in nursing homes, resulting in a unique retrospective cohort database. For this study we used data from two sources: (1) the medical statistics of the Swiss hospitals (MS) administered by the Federal Statistical Office (FSO), which registers all hospital discharges. Data collection is mandatory for all Swiss hospitals. The MS is an administrative data set used amongst others as a data base for the DRG financing system of the Swiss hospitals. The second data source (2) is the Structural Survey (SE), part of the census of Switzerland, a representative sample of around 200,000 people, which is collected every year. We obtained data from five waves of the SE (2010–2014). The data were collected and anonymized by the FSO using a hashing procedure. For a detailed description of the different data sources and the linkage procedure see (45). The database underwent comprehensive validation and the matched records were evaluated for completeness and correctness. There was no evidence of bias, because the subsample of hospitalizations successfully linked to individuals is to be regarded as a random selection of the study population with one exception: the under-representation of some non-European migration groups happening as a result of inconsistent writing of unfamiliar names. For details of the evaluation process, see (45). The *source population* of the SIHOS-study includes all individuals 15 years and older living in Switzerland in a private home, who were hospitalized (as inpatients) in an acute hospital at least one time between the years 2010 and 2016. The SIHOS *database population* consists of all individuals of the source population who participated in the public census in one of the Structural Survey waves between 2010 and 2014, for which a successful linkage between census data and medical data could be created.

### Study Sample

**Figure 2:**
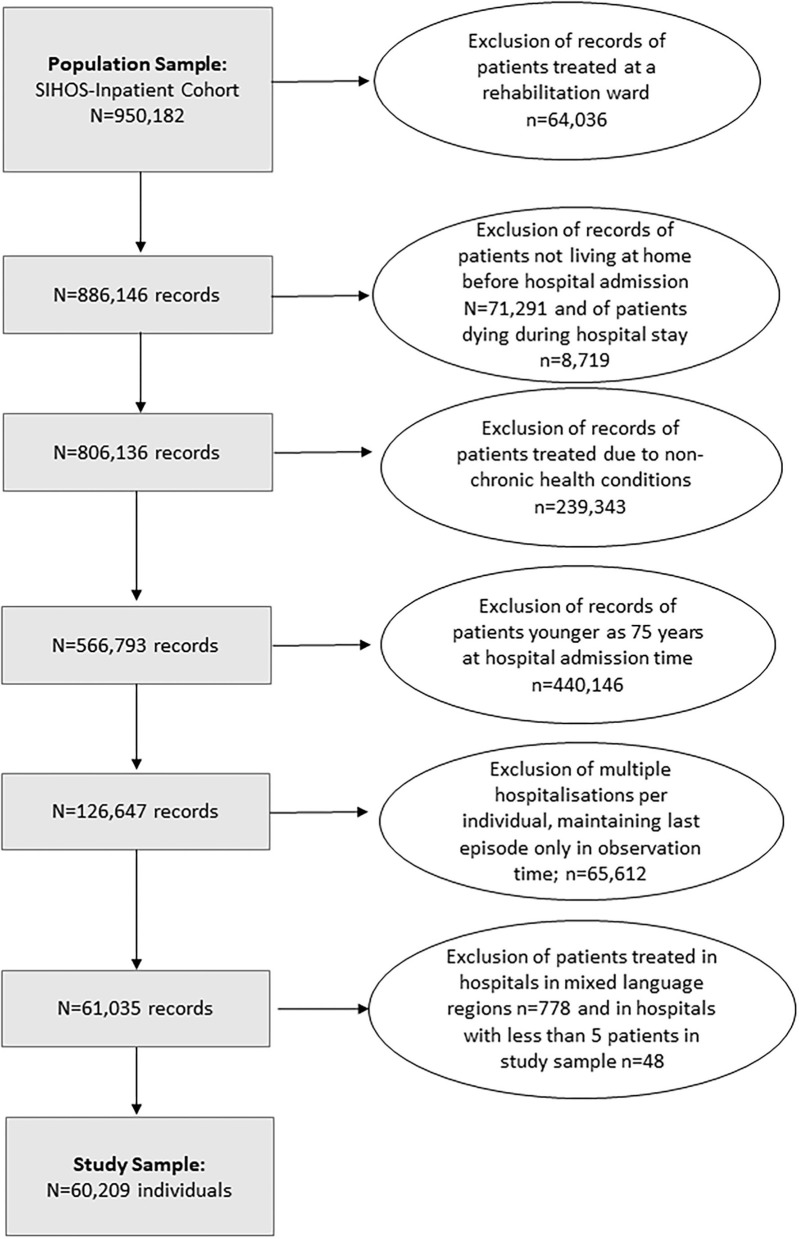
Flow chart of selection of cases to define the study sample (*N* = 60,209 individuals).

This study included individuals 75 years old and older at the time of hospitalization who had at least one acute hospital admission between 2010 and 2016 related to a chronic condition and who had been living at home before hospital admission. Excluded were patients who were admitted only to a rehabilitation ward, who died during their hospital stay or were hospitalized not due to a chronic health condition. In accordance with our question on the influence of the language regions, hospitals and their patients were excluded for which no clear identification of a language region could be made or in which fewer than 5 patients were represented in the study sample (for the selection of cases see flow chart, Figure 2).

For individuals with more than one acute hospitalization, the last acute hospital admission during observation time was included in the study. For our research question, the last hospitalization was of greater interest than e.g., the first one, because after a permanent nursing home admission, the individual drops out of the study sample. Following the logic of our sample selection, it is more probable that the last hospitalization during our observation time is the crucial moment when nursing home admission occurs. To control for the effect of multiple hospital stays of one individual the number of hospitalizations during the observation time was included as a covariate in the model. The sample defined in this way consisted of 60,209 individuals. The sample characteristics are described in Table 1.

**Table 1:** Descriptive characteristics of the study sample, *N*, percentages.

	**Admission to nursing home**		**Admission to other places**	
	***n***	**%**	***n***	**%**
Total sample	4,691	7.8	55,518	92.2
Gender				
Male	1,797	6.4	26,130	93.6
Female	2,894	9.0	29,388	91.0
Age groups				
75 to 79	813	3.4	23,381	96.6
80 to 84	1,299	6.8	17,941	93.2
85 and older	2,579	15.4	14,196	84.6
Nationality				
Swiss	4,404	8.0	50,565	92.0
EU/EFTA	266	5.5	4,567	94.5
Other states	21	5.2	384	94.8
Missing values	0	0.0	2	100.0
Education level				
Compulsory	2,256	9.2	22,171	90.8
Upper-secondary	1,929	7.2	24,682	92.8
Tertiary	506	5.5	8,665	94.5
Hospital insurance class				
Compulsory	3,516	8.7	36,680	91.3
Semi-private	793	6.1	12,187	93.9
Private	382	5.4	6,647	94.6
Missing values	0	0.0	4	100.0
Single household				
Lives with other people	1,969	5.6	32,891	94.4
Lives alone in household	2,722	10.7	22,627	89.3
Principal diagnosis				
Malignant neoplasms	666	9.9	6,036	90.1
Cardiovascular diseases	1,218	7.2	15,642	92.8
Chronic respiratory diseases	123	8.7	1,286	91.3
Diabetes	57	12.3	407	87.7
Musculoskeletal diseases	584	4.5	12,539	95.5
Mental disorders	415	22.9	1,401	77.1
Dementia-related disorders	352	39.9	531	60.1
Other chronic diseases	1,276	6.7	17,676	93.3
Number of somatic comorbidities elixhauser				
None	671	4.2	15,170	95.8
One	801	5.3	14,265	94.7
Two	970	8.0	11,218	92.0
Three to four	1,558	12.0	11,461	88.0
Five and more	691	16.9	3,404	83.1
Mental comorbidity	605	14.2	3,668	85.8
Dementia-related comorbidity	608	28.6	1,521	71.4
Language region of hospital				
German	3,163	8.3	34,791	91.7
French	1,105	6.5	15,902	93.5
Italian	423	8.1	4,825	91.9

### Variables

#### The Outcome Variable

The outcome variable for this analysis is (1) admission to a nursing home after hospital discharge vs. (0) discharge to another place. This information is assessed by the hospitals as part of the medical statistics, MS. Discharge to another place most often includes returning home, being transferred to a rehabilitation clinic or to a second acute hospital. The problem of a possible misclassification bias in the outcome variable in the form of specific patient paths is discussed in section Discussion (e.g., older individuals who lived at home being admitted to an acute hospital, then being discharged to a rehabilitation clinic and finally moving permanently to a nursing home after the rehabilitation treatment).

As *covariates* we include gender with (1) female (0) male; age at hospital admission in three categories (0) 75 to 79 years, (1) 80 to 84 years old and (2) over 85 years old; nationality with three categories (0 = Swiss nationality; 1 = EU / EFTA states; 2 = other nationalities); year of hospitalization with seven categories (2010 = 0; 2011 = 1; 2012 = 2; 2013 = 3; 2014 = 4; 2015 = 5; 2016 = 6).

#### Health Status

Health status is described using five variables. First, we used the *main diagnosis* of the acute hospitalization (ICD10-GM diagnosis codes registered in the acute hospitals) grouped according to Clinical Classification Software^2^ (CCS Level 1). Eight dummy variables (1 = disease present, 0 = not present) were included in the statistical model representing the following common chronic disease groups: (a) malignant neoplasms, (b) cardiovascular diseases, (c) chronic respiratory diseases, (d) diabetes, (e) musculoskeletal diseases, (f) mental disorders, (g) dementia-related disorders, taking into account the “dagger and asterisk” system in ICD-10 (considering symptoms of dementia as underlying disease and as manifestation) and (h) other chronic conditions. We then included three variables for the amount of comorbidities diagnosed in the hospitals taking into account up to ten additional diagnoses: (a) *number of somatic comorbidities* (46) in five categories, (0) none, (1) one, (2) two, (3) three to four, (4) five and more somatic comorbidities; (b) *mental comorbidity* (0 = none) (1 = yes) and (c) *dementia-related comorbidity* (0 = none) (1 = yes). For details of the grouping procedures, indicating the ICD codes used, see Supplementary Additional Table 1. Lastly, we controlled for the (d) number of hospitalizations before the index (=last) hospitalization grouped in the following categories: none (0), one (1), two (2), three to four (3) and five and more (4).

Three variables are included in the model as *predictor variables of the social situation*. As an indicator for educational attainment, the SIHOS database includes the *highest educational qualification* achieved in three categories, (0) tertiary level qualification (university or college degree), (1) upper secondary level qualification (mainly vocational education) and (2) compulsory education or less (corresponds with an educational duration of nine years). Educational attainment is a classic indicator of vertical social inequality and displays a strong and consistent relationship with risks of disease and mortality in a population (47). The variable *household type* with the two categories (1) living alone and (0) living with other people in a private household is used as an indicator for a person's social resources. Living alone does not preclude the possibility of having a large social network, but in the case of older people living alone it often means that there is a lack of immediate, everyday support at home, which is especially needed after a hospital discharge. Since there is no direct information on income in our data set, we use *hospital insurance class* with three categories, (0) private, (1) semi-private and (2) general insurance class as a proxy variable for financial resources. To validate this association, we analyzed the relationship between income classes in Switzerland and average expenditure on supplementary hospital insurance. The representative sample of the Household Budget Survey (FSO) showed a clear relationship between income class and expenditure for supplementary hospital insurance (private or semi-private insurance class) for individuals living alone and also for households with couples in the population 65 years old and older. There was a 3.4-fold increase in expenditure for individuals living alone and a 2.7-fold increase in expenditure for couples in the highest income class compared to the lowest^3^. Another new study shows that the Swiss population with private or semi-private hospital insurance have a higher income and a higher level of education compared to the population without supplementary insurance (48). We therefore assume that this variable may be used as proxy for the financial resources of the patients. At the same time, in its function as a financial incentive system, the insurance class seems to have an impact on the use of health care and type of treatment during hospital stay (48, 49).

#### Language Region of Hospitals

For data protection reasons, the SIHOS data set does not contain any geographical information on the hospital or individual level, but includes a variable assessing the main language spoken by the patients. Assuming that a hospital in which the majority of patients (70% and more) indicate German (including Romansh), French or Italian as their main language is located in the respective region, most hospitals can be assigned to the three main language regions. Seven out of 221 hospitals are in a mixed language region and were excluded from the study sample. The variable language region has three categories with (0) German-speaking part, (1) French-speaking part and (2) Italian-speaking part.

### Methods of Analysis

The SIHOS database was imported into the statistic software program SPSS (Version 26.0). Validation procedures were carried out in SPSS 26, descriptive analysis in SPSS 27. The frequency procedure was used to analyze the descriptive characteristics of the study sample. Absolute numbers as well as percentages were tabulated stratified for the outcome variable “admission to a nursing home” or “admission to another place.”

In order to take the hierarchical structure of the data into account, a multilevel analysis with 60,209 patients at level 1 nested in 199 hospitals at level 2 was applied. For the multilevel logistic regression analysis we used MlwiN, Version 3.01 from the Center for Multilevel Modeling, University of Bristol. The association between the independent social variables and the likelihood of discharge to a nursing home, adjusted for covariates and clustering effect on hospital-level, was analyzed entering the covariates and predictors in five blocks (see Table 1).

To estimate the parameters we used a quasi-likelihood approach based on Taylor series expansion as approximation to linearize the nonlinear function. Second order Taylor approximation or penalized quasi-likelihood (PQL) has proved to be a generally better estimation method than marginal quasi-likelihood (MQL) and first order Taylor approximation, especially if groups at lowest level are small and random effects are large (50). As statistic test Wald test is recommended, since it is a quasi-likelihood method and AIC, BIC as well as comparing deviances are not very accurate (51). As a condition for the application of a logistic regression analysis to avoid overfitting, Hosmer and Lemeshow state that the number of individuals in the outcome category with smaller n must be at least ten times the number of all variables included in the model. This condition is given in our analysis with 16 variables with a total of 50 dummy-coded categories in the full model and 4,691 individuals in the smaller outcome category. To test for possible collinearity between the independent categorial social variables education level, hospital insurance class and household structure, we used Cramer's V, providing the best balance between Type I error control and power (52). The results showed no critical association among the variables.

## Results

Table 2 describes the characteristics of the study sample. 7.8% or 4,691 of the total of 60,209 patients in the study sample were admitted directly to a nursing home after hospital discharge. In bivariate comparison, the rate of admission to a nursing home was higher for women, older patients, Swiss nationals, patients with a low education level, patients with no supplementary hospital insurance, and those living alone. The most important single characteristic is an indicator of health status: 39.9 percent of the patients with the main diagnosis of dementia are admitted to a nursing home, followed by 28.6 percent of patients with a dementia-related comorbidity and 22.9 percent of patients with mental disorder as the principal diagnosis.

**Table 2:** Overview of the multilevel logistic regression model building process.

**Model**	**Variables included**
A	Model with random intercept Including social predictors, adjusted for age, gender, nationality and year of hospitalization
B	Model A complemented by variables assessing the health status as covariates
C	Model B complemented by interaction terms between the social predictor variables
D	Model C complemented by main effect of language region of hospital
E	Model D complemented by interaction terms between the social predictor variables and language region of hospital.

In Table 3, we report the crucial parameter estimates from the different logistic regression models A, B and D; for details of all models see Supplementary Additional Tables 2, 3.

**Table 3:** Results of the multilevel logistic regression models A, B and D with random intercepts; Estimations for social predictors, health covariates and regional variables (p, odds ratio and 95% confidence interval for single predictors; joint Chi2 test (df), p and ICC (intra-class-variance); Models adjusted for gender, age group, nationality, year of hospitalization and number of hospitalizations before index-hospitalization.

	**Model A**				**Model B**				**Model D**			
		**95% CI**				**95% CI**				**95% CI**		
	**OR**	**Lower**	**Upper**	**Sig**.	**OR**	**Lower**	**Upper**	**Sig**.	**OR**	**Lower**	**Upper**	**Sig**.
**Individual level**		
Education level (ref.: tertiary)	1.00				1.00				1.00			
Upper-secondary	1.13	1.01	1.26	^*^	1.09	0.98	1.22		1.09	0.97	1.22	
Compulsory	1.22	1.09	1.37	^***^	1.15	1.03	1.30	^*^	1.16	1.03	1.30	^*^
Insurance class (Ref.: private)	1.00				1.00				1.00			
Semi-private	0.95	0.83	1.09		0.98	0.86	1.13		0.98	0.85	1.13	
Compulsory	1.19	1.06	1.35	^**^	1.24	1.09	1.41	^**^	1.24	1.09	1.41	^**^
Household (ref.: living with others)	1.00				1.00				1.00			
Single household	1.55	1.45	1.67	^***^	1.64	1.52	1.76	^***^	1.64	1.53	1.76	^***^
Principal diagnosis (ref.: other)					1.00				1.00			
Malignant neoplasms					1.80	1.62	2.00	^***^	1.80	1.62	2.01	^***^
Cardiovascular diseases					0.81	0.74	0.89	^***^	0.81	0.74	0.89	^***^
Chronic respiratory diseases					1.04	0.84	1.28		1.04	0.84	1.28	
Diabetes					1.24	0.91	1.70		1.24	0.91	1.70	
Musculoskeletal diseases					0.85	0.76	0.95	^**^	0.85	0.76	0.95	^**^
Mental disorders					4.67	4.01	5.44	^***^	4.73	4.06	5.50	^***^
Dementia-related disorders					11.75	9.91	13.93	^***^	11.95	10.07	14.17	^***^
Nr. of somatic comorbidities (ref. none)			1.00				1.00			
One					1.36	1.21	1.54	^***^	1.36	1.20	1.53	^***^
Two					1.91	1.69	2.15	^***^	1.90	1.68	2.14	^***^
Three to four					2.75	2.45	3.09	^***^	2.74	2.44	3.07	^***^
Five and more					4.27	3.72	4.90	^***^	4.25	3.70	4.88	^***^
Mental comorbidity (ref.: none)					2.39	2.16	2.65	^***^	2.39	2.16	2.65	^***^
Dementia-related comorbidity (ref.: none)					5.14	4.60	5.75	^***^	5.17	4.62	5.78	^***^
**Hospital-level**												
Language region (ref.: German-speaking)								1.00			
French-speaking region									0.54	0.37	0.80	^**^
Italian-speaking region									0.70	0.37	1.31	
Joint Chi2 (df), *p*	1,949.5 (16), *p* < 0.001				4,002 (32), *p* < 0.001				3,987.9 (34), *p* < 0.001			
ICC	34.4%				21.3%				20.0%			

* *p < 0.05*;

** *p < 0.01*;

*** *p < 0.001*.

**Figure 3:**
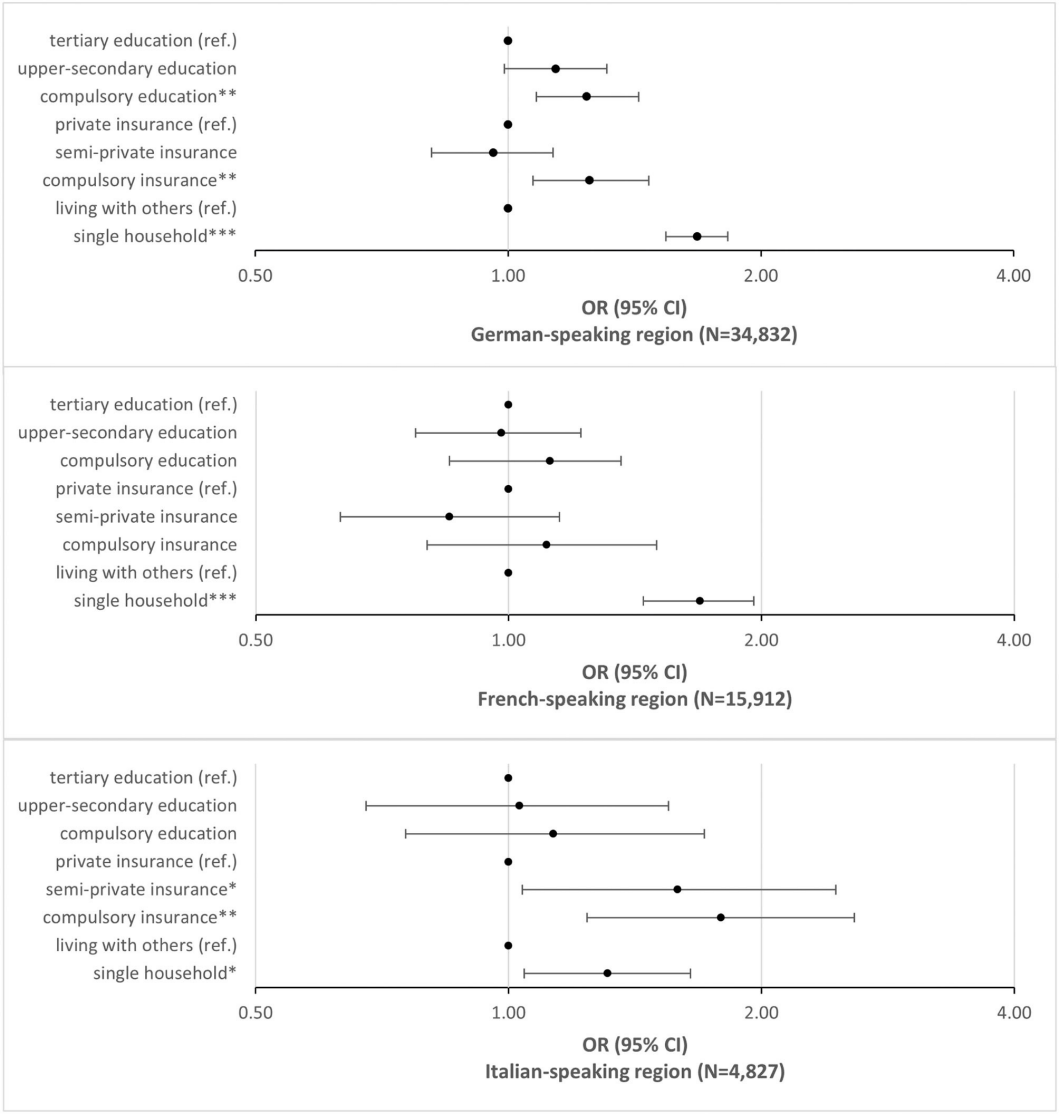
Social determinants and nursing home admissions in the language regions. Odds of admission to a nursing home after hospital discharge; logistic regression model B stratified for the three language regions in Switzerland controlled for gender, age group, nationality, health status and year of hospital admission: OR, CI 95%, *p < 0.05; **p < 0.01; ***p < 0.001.

The parameter estimates of **Model A** show significant associations between the three social predictors and the odds of being admitted to a nursing home, controlling for gender, age, nationality, year of hospitalization and hospital-level variance. Compared to individuals with tertiary education, those with compulsory (OR = 1.22) or with upper secondary education (OR = 1.13) have higher odds of moving to a nursing home after hospital discharge. The same applies to individuals without supplementary hospital insurance (OR = 1.19) compared to individuals with private insurance, the highest insurance class, whereas individuals with semi-private insurance do not differ from those with the private insurance class. The highest odds show individuals living alone compared to those living with others in a household (OR = 1.55). The between-hospital variance (ICC) of Model A is high with a proportion of 34.4% of the total variance. **Model B** includes the variables describing the health status of patients. Very strong and significant predictors for admission to a nursing home are the principal diagnosis of a mental disorder (OR = 4.67) and even more pronounced, a dementia-related disorder (OR = 11.75). Further, a high number of somatic comorbidities and mental and dementia-related comorbidities are strong predictors of nursing home admission. After controlling for these health-related covariates, the effects of the social variables remain significant, though reduced for education level, while the effect of insurance class and living alone vs. living with other persons becomes slightly more pronounced. The included parameters reduce the between-hospital variance markedly to 21.3%. In **Model C**, none of the tested interaction terms showed a significant effect, thus none of these terms were included for further model development (see the detailed results in the Supplementary Additional Table 3). Including the language regions in **Model D** showed a significant difference between the language regions of Switzerland: Taking into account the effect of social variables, health status and demography as well as the variance between the hospitals, the patients hospitalized in the French-speaking region had markedly lower odds of discharge to a nursing home (OR = 0.54) compared to the patients in the German- or Italian-speaking regions. Including the language regions as parameters in the model did merely reduce the level 2 variance. Finally, **Model E** tested the hypothesis that there is an interaction between the social predictors and the three main language regions that may explain a significant part of the variance of hospital admission. We found a relevant interaction effect between the different categories of insurance class and the language regions and therefore stratified Model B by language region (see Figure 3 and details in the Supplementary Additional Table 5).

The stratified analysis revealed that both socio-economic variables—educational attainment and insurance class—are moderated by the regional context, but not the effect of living alone. For the German-speaking region of Switzerland, the associations between all three social variables and the odds of admission to a nursing home remain significant. A low education level, having no private insurance and living alone are all associated with higher risks of moving to a nursing after hospital discharge. The result for the French-speaking part of Switzerland shows a different picture: living alone is a significant predictor of admission to a nursing home, but neither education level nor insurance class. In the Italian speaking part, living alone and insurance class are significant predictors of the outcome, but education level is not.

## Discussion

In this paper we have examined whether there are social inequalities in the likelihood of admission to nursing homes after an acute hospital stay in the older population with chronic conditions in Switzerland. Of the total of 60,209 patients in our study sample 7.8% or 4,691 were admitted directly to a nursing home after hospital discharge. We found significant effects of education, insurance class and living alone in a model adjusted for age, gender, nationality and year of hospitalization. Patients with the lowest education level had a 22% higher likelihood of being admitted to a nursing home compared to patients with the highest education level. Patients without supplementary insurance had a 19% higher likelihood of new institutionalization compared to patients with the highest insurance class (private). For individuals living alone this likelihood was 55% higher compared to older people living with others. Taking into account the health status of the patients, we found somewhat weaker but persisting effects of education level and stable effects of insurance class and living alone. This result is in line with our first hypothesis. Regarding the effect of the insurance class, our findings strengthen the evidence that financial resources are an important predictor of institutionalization in developed countries (10). Regarding the effect of education level the interpretation of our results are more complex. Including the different indicators of morbidity and comorbidity in our model reduces the effect of education on the odds of nursing home admission. We assume that this result is related to the fact that low educational resources increase the risk of developing chronic health conditions and also the risk of an exacerbation of these diseases. The stronger effect of education level found in model A would therefore be caused partly by the poorer health status of the individuals low in education. The complex and reciprocal relationship between lower education, higher risk of morbidity or multimorbidity and probability of a nursing home admission in old age may explain the somewhat inconclusive results concerning the effect of education in review studies (10). An other analysis based on the SIHOS database showed that patients with low education level hospitalized due to a chronic health condition more often are diagnosed with multimorbidity (53). This points to the multiple burden on this population group and the necessity of sufficient support during and after hospitalization. The result of a persistent and strong effect of living alone to predict new institutionalization after acute hospital discharge is consistent with the results found by Agosti et al. (30) in Italy and Harrison et al. (2) in Scotland. This similarity of the findings provides further support for a stable and consistent relationship between living alone and a higher admission rate to nursing homes rate independent of medical needs or SES like education or income. This result could stimulate the discussion about new forms of housing for older people (e.g., co-living arrangements).

In accordance with our second hypothesis (model D) we found a main effect of the language regions that were interpreted as indicators for the regional infrastructure and old age policies: In the French-speaking region the odds of being admitted to a nursing home after hospital discharge are 46% lower than in the German-speaking regions of Switzerland. This result is consistent with studies observing disparity in old age health care policies and infrastructure between the cantons and language regions in Switzerland (39, 54, 55). The interaction analysis between regions and socio-economic parameters revealed a significant effect for insurance class, but not for education level nor living alone (model E). To analyze this effect in more detail, the sample was stratified by language region and the models were calculated separately for each region. We found that our third hypothesis was only partially supported by our results:

The effect of the insurance class as proxy for income is clearly moderated by the regional context, the effect of education level is somewhat weaker moderated, whereas the effect of living alone is comparable in all three language regions. Individuals with fewer education-related and financial resources seem to be better off in the French-speaking part of Switzerland, where the older individuals with a lot or with few resources did not significantly differ as far as the question at stake is concerned. This may be explained by the stronger implementation of the ambulant care and the intermediate infrastructure (e.g., SPITEX and assistant living) and the accessibility of these services for the whole population in this region. Based on Beckfield's institutional theory of welfare states, we could therefore presume that the language regions in Switzerland are characterized by different meso-level arrangements that influence the social inequality in health and health care in their populations (22). However, this does not seem to be true for the aspect of the availability of social support at home: Living alone remains a stable risk factor for institutionalization in our study that is not mitigated by regional aspects.

### Strength and Limitations

One of the unique strengths of this study is the ability to link individual census data to medical inpatient data of all acute hospitals in Switzerland. Therefore the study sample provides a nearly representative sample of the older population treated as inpatient in an acute hospital. In comparison to many other studies the sample is not restricted to a specific fraction of health problems or a specific fraction of the hospital care.

The limitations are mainly related to the implications of routinely collected data not created to answer the study questions. As a consequence of this, we must assume the existence of unmeasured confounding. First of all, in order to conduct a comprehensive analysis of our hypothesis, we would have preferred to include information about the hospital discharge management at the hospital-level. Information on the involvement of social work in the preparation of hospital discharge and support for social disadvantaged patients would have been of particular interest. However, taking into account the hospital-level-variance did not change the main effects we found in our study. Second, we were unable to include information about the access to health and social care at the community or cantonal level because these geographical information were not included in the SIHOS-database. Unmeasured confounding is also possible in the assessment of the health status in our study. The medical data assessed in the hospitals records only health problems with a relevance for the medical care during the hospital stay. A further potential limitation of this study includes the use of one-time survey data to measure risk factors for a nursing home admission. Given the length of the follow-up time (in extreme cases, six years of difference), it is possible that these may have changed over time and thus produce a time-varying measurement bias. This problem concerns only the predictor variable 'living alone'. We assume that education level will hardly change in old age and the insurance class was assessed as part of the medical record at the time of the hospitalization. The bias concerning the predictor 'living alone' would result in a bias toward the null, therefore the observed effect size found in our study could be slightly underestimated.

Our outcome variable discharge to nursing home or to other places may include the problem of a misclassification bias concerning two specific patient paths: (a) patients being discharged to a rehabilitation ward and not returning home after discharge from rehabilitation, but instead moving to a nursing home. In the SIHOS database this specific patient path is rare (3.6% of all records of patients 75 years and older); most patients who are discharged from rehabilitation ward after an acute hospital stay return home. The second patient path of interest (b) includes patients using acute transition care (ATC) in a nursing home but returning home after a short time. Because ATC and short time stays of nursing home care were rarely used in Switzerland during the observation time of this study (ATC: 0.3% of all beds; Short time stays: 1.9% of all beds), we assume that misclassification bias is not a serious problem discharge (56).

### Conclusion

To conclude, the results of our study point to three aspects that are especially important for the promotion of social equality in health care for older people in Switzerland:

- Acute hospital discharge in older persons is a critical moment of transient dependency, in which social inequality affects the chance of living independently in one's own home.
- The effect of social determinants on nursing home admission after hospital discharge may be mitigated by the regional policy and health care.
- Future research should address the question which housing solutions, community services and primary health care interventions are effective in reducing the risk that socially disadvantaged older people will enter a nursing home without medical need.
- “Aging in place” as a key element of the quality of life and social integration should be accessible for all people regardless of income and place of living.

## Data Availability Statement

These data are the property of the Swiss Federal Statistical Office (SFSO) and can only be made available by legal agreements with the SFSO. The data that support the findings of the present analysis are used under license for the SIHOS study and are not publicly available due to the data protection restrictions. However, they are available after signing a data protection contract with the Swiss Federal Statistical Office (SFSO) Sektion Gesundheitsversorgung, Espace de l'Europe10, CH-2010 Neuchâtel, Switzerland Email: gesundheit@bfs.admin.ch.

## Author Contributions

NB participated in the development of the conceptualization and methodology and the acquisition of the funding of the SIHOS-study, designed and performed the analyses, interpreted the results, wrote the original draft of this paper, and edited the manuscript. AZ and LB-O participated in the development of the conceptualization and methodology and the acquisition of the funding of the SIHOS-study, interpreted the results, and edited the manuscript. All authors have read and approved the manuscript.

## Funding

This work was supported by the Swiss National Science Foundation, National Research Program Smarter Health Care (NRP74), Project Number 4, Grant Number 407440_167506. Project and funding description are available at http://www.nfp74.ch/en/projects/in-patient-care/project-bayer-oglesby. The funder had no role in the study design, data collection and analysis, decision to publish, or preparation of the manuscript.

## Author Disclaimer

The views reported here are the authors' views and do not necessarily reflect the funding organization.

## Conflict of Interest

The authors declare that the research was conducted in the absence of any commercial or financial relationships that could be construed as a potential conflict of interest.

## Publisher's Note

All claims expressed in this article are solely those of the authors and do not necessarily represent those of their affiliated organizations, or those of the publisher, the editors and the reviewers. Any product that may be evaluated in this article, or claim that may be made by its manufacturer, is not guaranteed or endorsed by the publisher.

## Footnotes

^1^ http://www.nrp74.ch

^2^CSS = Clinical Classifications Software, developed by the Healthcare Cost and Utilisation Project (HCUP), financed by the US Agency for Healthcare Research and Quality, adapted for Switzerland by Daniel Zahnd, BFH.

^3^Office Federal of Statistics, Household Budget Survey 2015-2017, Table T20.02.01.02.12.
