# Supplementary material for: Social and Regional Factors Predict the Likelihood of Admission to a Nursing Home After Acute Hospital Stay in Older People With Chronic Health Conditions: A Multilevel Analysis Using Routinely Collected Hospital and Census Data in Switzerland
Source: Front Public Health. 2022 May 9;10:871778. doi: 10.3389/fpubh.2022.871778 (PMC9126315; doi:10.3389/fpubh.2022.871778)
Supplement: Supplementary file 1 [file Data_Sheet_1.docx]

**Supplementary information**

Additional Table 1: Definition of the specific diseases based on Clinical Classification System Level 1 and ICD10-GM Codes (Version 2017)

| **Specific diseases** | **CSS Level 1*** | **ICD10-GM Codes (2017 version)** |
| --- | --- | --- |
| **Chronic somatic diseases** | | |
| Malignant neoplasms (cancer) | | |
| Lung cancer | CSS_LEV1 = 19 | C34, D022 |
| Colon cancer | CSS_LEV1 = 14 | C18, C010 |
| Breast cancer (women) | CSS_LEV1 = 24 | C50, D05 |
| Prostate cancer (men) | CSS_LEV1 = 29 | C61, D075 |
| Cardiovascular diseases (incl. risk factors) | | |
| Diabetes |  | E10–E14, R73 |
| Congestive heart failure (CHF) | CSS_LEV1 = 108 | I50 |
| Acute myocardial infarction (AMI) | CSS_LEV1 = 100 | I21,I22 |
| Acute cerebrovascular diseases | CSS_LEV1 = 109 | I60–I64, I66 |
| Chronic respiratory diseases | | |
| Chronic obstructive pulmonary disease (COPD) | CSS_LEV1 = 127 | J40–J44, J47 |
| Asthma | CSS_LEV1 = 128 | J45, J46 |
| Musculoskeletal diseases | | |
| Osteoarthritis | CSS_LEV1 = 203 | M15-M19 |
| Back problems | CSS_LEV1 = 205 | M43.2, M43.5, M43.6, M45, M46 (excl. M46.2, M46.3), M47, M48 (excl. M48.5) |
| **Mental disorders** | |  |
| Affective disorders | CSS_LEV1 = 69 | F3, F41.2 |
| Psychotic disorders | CSS_LEV1 = 70, 71 | F2, R41.0 |
| Alcohol-related behavioural disorders | CSS_LEV1 = 66 | F10, G31.2, R78.0 |

*CSS = Clinical Classifications Software, developed by the Healthcare Cost and Utilisation Project (HCUP), financed by the US Agency for Healthcare Research and Quality, adapted for Switzerland by Daniel Zahnd, BFH.

Additional Table 2: Results of the multilevel logistic regression **models A, B and D** with random intercepts; fixed effects: odds ratios, 95% CI and p for single predictors, joint Chi2 test (df), p; random part: variance, SE, Intra-Class-Correlation ICC

Additional Table 3: Results of the multilevel logistic regression **model C** with random intercepts; fixed effects: odds ratios, 95% CI and p for single predictors, joint Chi2 test (df), p; random part: variance, SE, Intra-Class-Correlation ICC

Additional Table 4: Results of the multilevel logistic regression **model E** with random intercepts; fixed effects: odds ratios, 95% CI and p for single predictors, joint Chi2 test (df), p; random part: variance, SE, Intra-Class-Correlation ICC

Additional Table 5: Results of the multilevel logistic regression **model B stratified for the language regions** with random intercepts; fixed effects: Odds ratios, 95% CI and p for single predictors, joint Chi2 test (df), p; random part: variance, SE, Intra-Class-Correlation ICC; (*) Model for italian speaking region could only be converged using first order MQL approximation.
